# Unraveling the genetic structure of Brazilian commercial sugarcane cultivars through microsatellite markers

**DOI:** 10.1371/journal.pone.0195623

**Published:** 2018-04-23

**Authors:** João Ricardo Vieira Manechini, Juliana Borges da Costa, Bruna Turcatto Pereira, Luciana Aparecida Carlini-Garcia, Mauro Alexandre Xavier, Marcos Guimarães de Andrade Landell, Luciana Rossini Pinto

**Affiliations:** 1 Departamento de Genética e Melhoramento de Plantas, Faculdade de Ciências Agrárias e Veterinárias (FCAV), Universidade Estadual Paulista “Júlio de Mesquita Filho”, Jaboticabal, SP, Brasil; 2 Centro de Cana, Instituto Agronômico de Campinas (IAC), Ribeirão Preto, SP, Brasil; 3 Agência Paulista da Tecnologia dos Agronegócios (APTA), Polo Regional Centro Sul, Piracicaba, SP, Brasil; USDA-ARS Southern Regional Research Center, UNITED STATES

## Abstract

The Brazilian sugarcane industry plays an important role in the worldwide supply of sugar and ethanol. Investigation into the genetic structure of current commercial cultivars and comparisons to the main ancestor species allow sugarcane breeding programs to better manage crosses and germplasm banks as well as to promote its rational use. In the present study, the genetic structure of a group of Brazilian cultivars currently grown by commercial producers was assessed through microsatellite markers and contrasted with a group of basic germplasm mainly composed of *Saccharum officinarum* and *S*. *spontaneum* accessions. A total of 285 alleles was obtained by a set of 12 SSRs primer pairs that taken together were able to efficiently distinguish and capture the genetic variability of sugarcane commercial cultivars and basic germplasm accessions allowing its application in a fast and cost-effective way for routine cultivar identification and management of sugarcane germplasm banks. Allelic distribution revealed that 97.6% of the cultivar alleles were found in the basic germplasm while 42% of the basic germplasm alleles were absent in cultivars. Of the absent alleles, 3% was exclusive to *S*. *officinarum*, 33% to *S*. *spontaneum* and 19% to other species/exotic hybrids. We found strong genetic differentiation between the Brazilian commercial cultivars and the two main species (*S*. *officinarum*: ${\hat{\Phi }}_{ST}$ = 0.211 and *S*. *spontaneum*: ${\hat{\Phi }}_{ST}$ = 0.216, P<0.001), and significant contribution of the latter in the genetic variability of commercial cultivars. Average dissimilarity within cultivars was 1.2 and 1.4 times lower than that within *S*. *officinarum* and *S*. *spontaneum*. Genetic divergence found between cultivars and *S*. *spontaneum* accessions has practical applications for energy cane breeding programs as the choice of more divergent parents will maximize the frequency of transgressive individuals in the progeny.

## Introduction

Sugarcane has a worldwide economic importance, with Brazil being the largest producer, harvesting over 657.2 million tons of sugarcane in 2016/17 [1]. In addition to its importance as a food, sugarcane is increasingly more important for ethanol production, and electricity cogeneration currently accounts for 4% of Brazilian electric energy demand [2]. Brazil’s position as a world leader in sugarcane production is attributed to not only climate conditions and adequate agronomical management practices, but also to the high performance of Brazilian commercial sugarcane cultivars, which have been developed by the country’s three main sugarcane breeding programs (RIDESA: Interuniversity Network for the Development of the Sugarcane Sector; CTC: Sugarcane Technology Center; IAC: Agronomic Institute of Campinas).

Sugarcane belongs to the genus *Saccharum*, related to other genera as *Erianthus* sect. *Ripidium*, *Miscanthus* sect. *Diandra* Keng *Narenga* Bor, and *Sclerostachya* Hack A. Camus, which grouped together, constitute the *Saccharum* Complex [3].

A major breakthrough for sugarcane came with the discovery that the *Saccharum* genus occasionally yields viable offspring with superior phenotypes via interspecific crosses [4]. Artificial interspecific hybridizations started between the 19^th^ and 20^th^ century in Asia by Dutch cane breeders [5] and basically, two main species were used in this approach: *S*. *officinarum*, known as “noble cane”, a decaploid (2n = 80, x = 10) with thick, juicy stalks rich in sugar but highly susceptible to several biotic and abiotic stresses, and *S*. *spontaneum*, an octaploid (2n = 40–128, x = 8) with no significant sugar content but highly resistant, vigorous [6,7], and efficient in carbon fixation due to its high photosynthetic rate [8]. The first interspecific hybrids were repeatedly backcrossed to *S*. *officinarum* accessions to recover the sucrose content in a process known as nobilization [5]. Therefore, approximately 80% of the genome of current sugarcane cultivars came from this ancestor species, 10–15% from *S*. *spontaneum* and the remaining 5–10% being recombinant chromosomes [9]. Other *Saccharum* species such as *S*. *robustum*, *S*. *barberi* and *S*. *sinense*, although used less extensively in the first artificial interspecific hybridizations, also contributed to the genome of modern sugarcane cultivars [10]. It should be noted that sugarcane cultivars from the first interspecific hybridizations have impacted the sugarcane industry of the twentieth century, especially the cultivar POJ2878, known as the “wonder cane” and genetically present in the majority of modern sugarcane cultivars [11]. Despite the polyploid nature of sugarcane and the great genetic variability available in the *Saccharum* germplasm, very few accessions took part in the first interspecific hybrids [12,13] leading to the narrow genetic base of the actual cultivars.

According to the International Union for Conservation of Nature (IUCN), the conservation of genetic diversity is crucial for the maintenance of biodiversity [14,15]. For instance, the exhaustive selection of certain traits over several breeding generations inevitably tends to threaten other traits. Certain genic regions or even alleles and gene subsets can be lost with time. Variability loss and genetic base narrowing directly implies biodiversity loss and intensifies the risk of susceptibility to an unexpected disease or pest occurrence [16]. To preserve genetic variability and biodiversity, germplasm banks are maintained by breeding programs for the conservation of genetic resources for use in future crossings, especially in genetic introgression programs [17]. There are several sugarcane germplasm banks in the world, such as the World Collection of Sugarcane and Related Grasses (WCSRG) in Miami, FL, USA and the Research Centre of Kannur in Coimbatore, India. In Brazil, one of the biggest germplasm banks in the world is maintained by CTC in Camamu, BA, Brazil [18]. These banks encompass a huge amount of accessions of *Saccharum* species and related genera including those collected in the sugarcane first expeditions, acting on an internationally collaborative level [19].

Undoubtedly, the genetic variability present in most of the germplasm banks is underexploited. Sugarcane germplasm banks have thousands of accessions, which complicate logistics and planning of future crosses. Organization and classification are key factors for the functionality of germplasm banks. Although morphological descriptors have an important role in accession classification and organization, errors may occur once environmental conditions affect phenotypes. Obtaining the molecular profile of the accession, which is a more reliable way to identify a given individual [20,21], is a complement to its morphological description. In addition, several molecular markers have been applied to investigate and measure the genetic diversity at the molecular level of sugarcane accessions and also to trace genetic relationship among *Saccharum* species [22,23]. Microsatellites or Single Sequence Repeats (SSRs) are among the most suitable and preferred markers for genetic structure and conservation studies [24]. SSRs are stretches of DNA composed by motifs of one to six base pairs repeated in tandem and flanked by conserved sequences, allowing the amplification through PCR by specific primer pairs [25]. In addition, SSRs are highly polymorphic and reproducible codominant markers, transferable among closely related species and genera [26]. Due to the ploidy nature of sugarcane, microsatellite markers are considered dominant markers once the presence of a given amplified product doesn’t account for its dosage [27]. SSRs are promising to establish unique fingerprints for cultivar identification [28]. For sugarcane, a large amount of SSR primer pairs is available for research, either developed from genomic enriched libraries such as those from the International Sugarcane Microsatellite Consortium (ISMC) [29] or from expressed sequence tags (EST) data mining (e.g., SUCEST–Sugarcane Expressed Sequence Tag Database), which are being extensively applied for genetic mapping, diversity studies and fingerprinting [30–32].

The narrow genetic base of sugarcane breeding programs and its implications for future sugar yield and productivity gains are concerns among sugarcane breeders around the world [33,34]. However, there is still a considerable genetic variability among the actual sugarcane cultivars [35]. Therefore, investigation into the genetic structure of current commercial cultivars, and the comparison of this structure with the gene pool of the main ancestor species (*S*. *officinarum* and *S*. *spontaneum*), permit breeders to better manage their crosses and germplasm banks.

The characterization of sugarcane basic germplasm accessions has gained attention because of its utilization by sugarcane breeding programs [10], mainly by those focused on energy cane cultivars development in which *S*. *spontaneum* has been used as parental in crosses with commercial cultivars to meet the demand for biomass production [36].

Due to the importance of the sugar and ethanol sector in the Brazilian economy, new sugarcane cultivars are release almost every year by the breeding programs increasing the number of cultivars available for the cane growers. However, the assessment of its genetic structure and hence, the narrow genetic base’s impact on the magnitude of the Brazilian sugarcane genetic breeding pool has not yet been completely investigated.

In the present study, the genetic structure of a panel of Brazilian commercial cultivars currently in use by cane growers (which in turn represents the actual gene pool of Brazilian sugarcane breeding programs) was assessed through microsatellite markers and contrasted against a group of basic germplasm composed mainly of *S*. *officinarum* and *S*. *spontaneum* accessions. Here, we also report a set of twelve SSR primer pairs that, taken together are capable to distinguish sugarcane commercial cultivars and basic germplasm accessions, showing high power in capturing genetic variability and hence allowing the application of molecular markers in a fast and cost-effective way for routinely cultivar identification and management of sugarcane germplasm banks. We found significant contribution of *S*. *spontaneum* in the genetic variability of Brazilian sugarcane cultivars. Nonetheless, our data suggest that there is still potentially exploitable allelic richness (approximately 85% *S*. *spontaneum* exclusive alleles were not accounted into commercial cultivars) that can be incorporated in the gene pool of Brazilian breeding programs through energy cane cultivar development. This is the first report to investigate the genetic diversity at the molecular level in a huge group of currently planted Brazilian sugarcane cultivars.

## Materials and methods

### Plant material

A total of 137 genotypes encompassing 81 Brazilian commercial sugarcane cultivars and 56 basic germplasm accessions (19 *S*. *officinarum*, 26 *S*. *spontaneum* and 11 genotypes comprising five *Saccharum* spp. exotic hybrids and six accessions representing other species—*S*. *barberi*, *S*. *robustum* and *Miscanthus* spp.) were collected from the IAC Sugarcane Germplasm Collection maintained at the Sugarcane Center, in Ribeirão Preto, SP, Brazil. For clarity, the last group of 11 genotypes will henceforth be referred to as “other species/exotic hybrids.” Most of the *S*. *spontaneum* accessions and other species/exotic hybrids was imported from the USDA’s World Collection of Sugarcane and Related Grasses (Miami, FL, USA). The 56 accessions, which compose the basic germplasm group, were previously selected, taking into consideration molecular data, as the most divergent, non-redundant and historically relevant accessions of the IAC Fingerprint SSR-based Data Bank, among over 300 others. Some of the historically relevant individuals [13,37] were also chosen according to its contribution in the nobilization process. Clones such as KASSOER, COIMBATORE and CHUNNEE were intensively used in early interspecific hybridizations and are present in the pedigree of several commercial cultivars worldwide, as well as the cultivar POJ2878, because of its remarkable contribution on the development of most of the current cultivars throughout the world [11,38]. A detailed list of all materials is found in S1 Table.

### DNA extraction and PCR amplification

Total genomic DNA was extracted from young leaves or leaf roll tissue according to the CTAB method [39]. PCR reactions were conducted in a 16 μl final volume containing 30 ng of template DNA, 0.2 μM of each forward and reverse primers (labeled with infrared dyes IR700 or IR800), 100 μM of each dNTP, 2.0 mM MgCl_2_, 10 mM Tris-HCl, 50 mM KCl, and 0.5 unit Taq DNA polymerase (Invitrogen, São Paulo, Brazil). Reactions were amplified on a MyCycler Thermal Cycler (BIO-RAD®) thermocycler. For CV (CanaVialis) primer pairs: CV29, CV37, CV38, CV60, CV79, CV94, and CV106 [28,30] thermocycling conditions were performed according to Palhares et al. (2012) [40]. For the SC (Sugarcane) primer pairs: SCB213, SCB312, SCB381, SCB423 and SCC436 [22,41] thermocycling conditions were as follows: denaturation at 94.0°C for 3 min followed by 30 annealing cycles (94.0°C for 1 min, annealing temperature specific for each primer for 1 min; extension of 72.0°C for 1 min) and a final elongation step at 72.0°C for 5 min. SSR primer pair sequences with respective annealing temperatures and expected allele range are listed in Table 1. Amplification products were separated by electrophoresis on 6% denaturing polyacrylamide gel on a NEN4300 DNA Analyzers (LI-COR Biosciences®). The attributes of the SSR primers as genetic markers (ubiquity, co-dominance and high heterozygosity) [42], allied to its ability in successfully identifying sugarcane genotypes [43] and also the low cost of the technique (in comparison to SNPs, for instance) was considered in their choices.

**Table 1:** Relation of SSR primer pairs.

SSR	Annealing Temp.	Repeat Motif	Forward Sequence (5´- 3´)	Size range (bp)
			Reverse Sequence (5´- 3´)	
**CV29**^**a**^	60.0°C	(ATCT)_14_	TCGCGTCCACCAATGTAACC	82–170
			GCGTGCATCGCTTGTGTCTT	
**CV37**^**a**^	60.0°C	(TTTC)_15_	GGATGGACGACGTGTCCTGG	106–177
			ATAAAGTGGCCGCTTGGATTGA	
**CV38**^**a**^	60.0°C	(CTTTT)_18_	GAAGCAGGGGCCTCAAGTTG	96–210
			GTCAAACAGGCGATCTGGCTC	
**CV60**^**a**^	60.0°C	(CTCTCC)_5_	AATCTGCACCCTGCCCTCTC	158–194
			CAGCTGGAGCATGGATGGAG	
**CV79**^**a**^	60.0°C	(CTATAT)_11_(TATAGA)_6_	GGCACTGCTGGTGGTTGATTG	131–231
			TCCCACATCAAGAGGCAGCTA	
**CV94**^**a**^	60.0°C	(AAAAAG)_5_(CGT)_5_	GGCAGGCCAAGATGAATGAAG	182–234
			AGCACAGCGGAGGGTACGG	
**CV106**^**a**^	60.0°C	(GGC)_8_	AAACAGAGCATACTCGAGGCC	78–162
			ACGTTGCTGACGAGGTTTTCC	
**SCB213**^**b**^	62.0°C	(TCC)_6_	AGCCGTCAGGGGTCAGG	247–337
			ATTCGATGGAGCCTGAGTGAG	
**SCB312**^**b**^	64.7°C	(TCC)_7_	AGTCCGTCGCCGTAATCATCTTG	190–278
			GCACCTCCTCCTTTCCTTCCTTATT	
**SCB381**^**b**^	60.0°C	(TAC)_8_	TGGAGCTCCGTCTTCTTGTT	190–257
			GCTAGCCCGTACATTGGGTA	
**SCB436**^**b**^	60.0°C	(GAG)_5_	AGTACGCCTGAGTCCTGACG	158–274
			AGGTGCAAGGGCTGATAGAA	
**SCC423**	AVAILABLE UNDER REQUEST			

Annealing temperatures, repeat motifs, forward and reverse primers (5’ to 3’), and size range of amplified products (in base pairs).

^a^ According to Maccheroni et al. (2009) [28]

^b^ Marconi et al. (2011) [22].

### Genotyping

Due to the polyploid nature of sugarcane, the amplified products were scored as presence (1) or absence (0). The 50-350pb molecular weight standard (LI-COR Biosciences®) was used to calibrate the sizes of the amplified alleles on each run. The gel images were manually scored and revised with Saga GT™ software (LI-COR Biosciences®). This software identifies the amplified product size to build the genotyping matrix.

### Data analysis

The Polymorphism Information Content (*PIC*), Effective Multiplex Ratio (*E*), Marker Index (*MI*), Resolving Power (*Rp*) and the percentage of different profiles (haplotype assessment) were taken as indexes to evaluate the efficiency/discriminatory potential of the 12 SSRs primer set to capture polymorphism and distinguish sugarcane genotypes. The percentage of different profiles refers to the different molecular patterns (fingerprints) generated by each SSR primer pair considering the total number of genotyped individuals with no missing data point. Furthermore, for each SSR primer pair, the frequency of each type of allele and the presence of exclusive alleles (private alleles) were also investigated.

Polymorphism Information Content (*PIC*) was calculated [44] according to the expression $PIC=1-\sum_{i=1}^{n}p_{i}^{2}-\sum_{i=1}^{n}\sum_{j=i+1}^{n}{2p}_{i}^{2}p_{j}^{2}$ (in which *p* is the frequency of the *j*^*th*^ pattern for marker *i*, and *n* is the number of alleles) using the *PIC Calculator* program [45]. The Multiplex Ratio (*n*) was estimated as the average number of fragments amplified per genotype while the Effective Multiplex Ratio (*E*) was estimated by the expression *E* = *nβ* in which *n* is the multiplex ratio and *β* the fraction of polymorphic loci [46]. The Marker Index (*MI*) was obtained by the expression *MI* = *PICxE*. The percentage of different profiles refers to the different molecular patterns (fingerprints) generated by each SSR primer pair. The Resolving Power (*Rp*) for each primer pair was calculated from the genotyping matrix by using the expression *Rp* = ∑*Ib*, where *Ib* is the allele information, i.e., *Ib* = 1 − (2 × |0.5 − *M*|), and *M* the proportion of the total genotypes containing the allele [47].

The frequency of the different allele types (allele frequency) observed for a SSR primer pair was estimated by dividing the number of times that each type of allele occurs by the total number of alleles amplified by the respective SSR primer pair. Genetic diversity (*D*) was estimate for each group of species and commercial cultivars according to the expression $D=1-\sum_{i=1}^{n}P_{ij}^{2}$, in which *P*_*ij*_ is the frequency of the *j*^*th*^ pattern for marker *i*, summed across *n* patterns [48]

The analysis of molecular variance (AMOVA) was performed by the Arlequim v.3.5 software [49] to quantify the degree of differentiation and distribution of the genetic variability between and within predefined groups: Brazilian cultivars and basic germplasm; Brazilian cultivars and *S*. *officinarum*; Brazilian cultivars and *S*. *spontaneum*; *S*. *officinarum* and *S*. *spontaneum*.

The number of sub-populations (k) and the membership proportion (Q) was inferred by using Structure v.2.3.4 software [50,51]. The k was set from 1 to 10 (k-value), with 10 iterations at a 100,000 burning period and 200,000 Markov Chain Monte Carlo (MCMC) repeats [10]. Values of k and ΔK were obtained by Structure Harvester [52]. Best values for k were confirmed by the CLUMPAK Best k pipeline [53].

The pair-wise dissimilarity was estimated based on the Jaccard similarity coefficient complement (Dissimilarity = 1 –Similarity) [41,54]. The phylogenetic tree was constructed according to the Neighbor-Joining method with 1,000 bootstrapping using the DARwin v.6.0.13 software [55,56] also for the identification of the extreme dissimilarity values. To verify if the number of markers used to estimate the genetic dissimilarities between accesses was adequate in terms of accuracy, the bootstrap resample technique [57] was applied as in Tivang et al. (1994) [58] and Hállden et al. (1994) [59]. The binary matrix of the genotypes was resampled, considering different numbers of markers (in the present research, 3, 6, 9, …, 285 markers). For each of the 95-sample size considered, 1,000 bootstraps were performed, and for each bootstrap sample, a dissimilarity matrix was obtained, allowing for the calculation of the average coefficient of variation (CV%) of the estimates for each sample size. An exponential function was adjusted to estimate the number of markers needed to assure that the CV associated with the dissimilarity estimates were lesser or equal to 10%—considered acceptable in this research. The median (not the mean) of the coefficient of variation estimates were used to evaluate the accuracy of the dissimilarity values [60]. All these procedures were performed in RStudio software [61] as in Urashima et al. (2017) [62].

Aiming to detect the presence of some clustering pattern among the evaluated genotypes, Principal Coordinate Analysis (PCoA) [63–65] was conducted based on the dissimilarities between each pair of accessions.

## Results

### Analysis of SSR primer pairs efficiency

The 12 SSR primer pairs generated 285 alleles (markers) among the 137 genotypes. This set of SSR primers produced high quality fingerprints (S1 Fig), minimizing genotyping errors. The number of alleles ranged from 10 (CV60) to 43 (SCC423), with an average of 23.75 alleles per primer pair (locus) (Table 2).

**Table 2:** SSR Primer pair efficiency.

SSR	N	$\hat{PIC}$	$\hat{E}$	$\hat{MI}$	$\hat{RP}$	Identified profiles (%)
		All / C	All / C	All / C	All / C	
**CV29**	23	0.906 / 0.913	7.387 / 8.272	6.800 / 7.552	10.478 / 8.938	131 (95.6%)
**CV37**	20	0.879 / 0.878	5.234 / 6.012	4.692 / 5.278	7.119 / 6.074	125 (91.2%)
**CV38**	27	0.934 / 0.903	6.693 / 7.037	6.218 / 6.357	10.94 / 7.654	134 (97.8%)
**CV60**	10	0.788 / 0.720	2.956 / 2.938	2.262 / 2.115	3.149 / 2.568	41 (29.9%)
**CV79**	27	0.901 / 0.874	5.949 / 5.951	5.358 / 5.203	7.448 / 5.877	125 (91.2%)
**CV94**	14	0.776 / 0.734	3.781 / 3.605	3.185 / 2.646	4.896 / 1.778	52 (38.0%)
**CV106**	14	0.809 / 0.848	4.175 / 4.519	3.616 / 3.831	6.746 / 5.605	88 (64.2%)
**SCB213**	30	0.919 / 0.883	5.569 / 4.728	5.148 / 4.175	9.746 / 7.284	131 (95.6%)
**SCB312**	29	0.906 / 0.922	7.044 / 7.531	6.565 / 6.946	11.746 / 10.642	133 (97.1%)
**SCB381**	24	0.894 / 0.916	7.255 / 8.457	6.668 / 7.743	9.761 / 8.543	128 (93.4%)
**SCB436**	24	0.818 / 0.850	4.628 / 5.235	3.973 / 4.448	4.955 / 2.889	82 (59.9%)
**SCC423**	43	0.925 / 0.906	6.285 / 5.765	5.854 / 5.222	11.000 / 8.198	133 (97.1%)
**Total**	285					
**Mean**	23.75	0.871 / 0.862	5.580 / 5.837	5.028 / 5.126	6.914 / 6.337	

Total number of alleles (N), estimates of Polymorphism Information Content (PIC), Effective Multiplex Ratio (E), Marker Index (MI), Resolving Power (Rp) and number of identified profiles among 137 individuals (“All” stands for the results for all 137 accessions; “C” stands for the results considering only the group of cultivars).

PIC estimates varied from 0.776 (CV94) to 0.934 (CV38), with a mean value of 0.871 for all the 137 accessions, and for the cultivars, from 0.720 (CV60) to 0.922 (SCB312), with mean of 0.862. The Effective Multiplex Ratio estimated values varied from 2.956 (CV60) to 7.387 (CV29), with mean of 5.580 for all accessions, and from 2.938 (CV60) to 8.457 (SCB381), with mean of 5.837 for the cultivars. The Marker Index estimate values ranged from 2.262 (CV60) to 6.800 (CV29), with mean of 5.028 for all accessions, and from 2.115 (CV60) to 7.743 (SCB381), with mean of 5.126 for the cultivars.

To assess the ability of the primer pair for distinguishing among genotypes, the Resolving Power was calculated for the total of genotypes or for the cultivars only. For total genotypes, the Resolving Power estimates ranged from 3.149 (CV60) to 11.746 (SCB312), while for the cultivars it varied from 1.778 (CV94) to 10.642 (SCB312).

The highest index values for PIC, Effective Multiplex Ratio, Marker Index and Resolving Power across all accessions was obtained for primer pairs CV38 (0.934), CV29 (7.387), CV29 (6.800) and SCB312 (11.746) respectively. Considering only the group of cultivars, the highest index values for PIC, Effective Multiplex Ratio, Marker Index and Resolving Power were obtained for primer pairs SCB312 (0.922), SCB381 (8.457), SCB381 (7.743) and SCB312 (10.642) respectively. Primer pair CV38 generated the highest number of different profiles (134 profiles, 97.8%) for the whole 137 accessions (Table 2).

Haplotype assessment revealed that eight primer pairs (CV29, CV37, CV38, CV79, SCB213, SCB312, SCB381, and SCC423) were able to produce different SSR profiles in more than 90% of the individuals while CV106 in 64.2% and SCB436 in 59.9%. The lowest percentage of different profiles (38.0% and 29.9%) was obtained for CV94 and CV60 respectively.

### Allele frequency distribution, exclusive alleles, and genetic diversity

**Fig 1:**
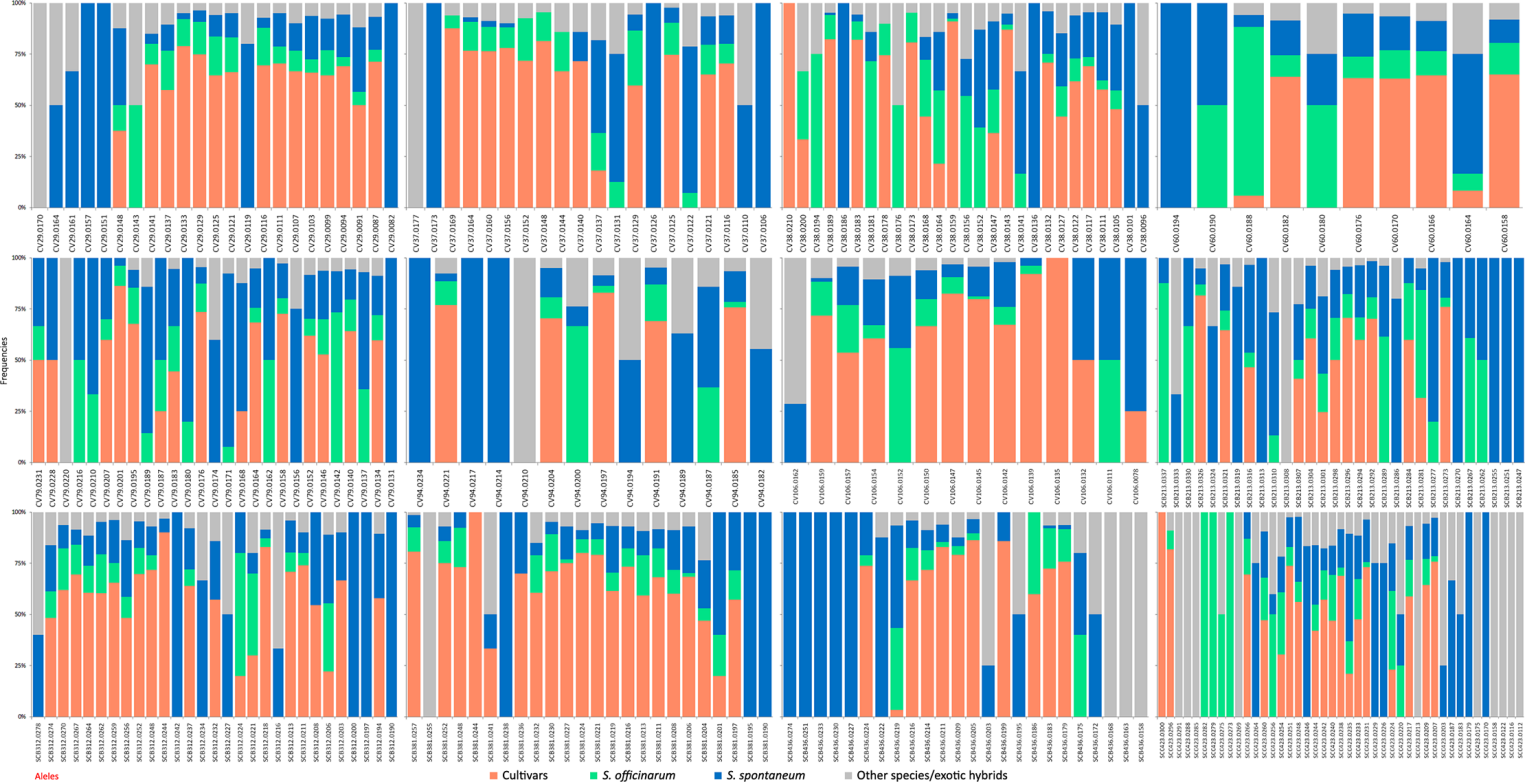
Allelic distribution for each primer pair.

The number of alleles observed for the Brazilian commercial cultivars, *S*. *officinarum*, *S*. *spontaneum*, and other species/exotic hybrids was 166, 186, 246 and 215, respectively (Fig 1). Among the basic germplasm group, four (1.4%), 39 (13.68%) and 19 (6.67%) were the respective putative exclusive alleles of *S*. *officinarum*, *S*. *spontaneum*, and other species/exotic hybrids.

Four alleles (SCB381.0244, SCC423.0300, CV38.0210, and CV106.0135), representing 1.4% of the total alleles, were exclusive to the commercial cultivars. One hundred and nineteen alleles (41.8%) spread across the basic germplasm accessions were not present in the Brazilian cultivars. Of these, 55 putative exclusive alleles of the basic germplasm group (three from *S*. *officinarum*, 33 from *S*. *spontaneum* and 19 from other species/exotic hybrids) were not found in the Brazilian cultivars (S2 Table).

To find the species origin of the alleles present in the Brazilian commercial cultivars, the presence of putative exclusive alleles from *S*. *officinarum*, *S*. *spontaneum*, and other species/exotic hybrids were searched for in the Brazilian cultivars. Thus, based on these putative exclusive alleles previously identified, three commercial cultivars (CTC10, RB865230, SP841431) showed one putative exclusive allele (SCB436.0186) derived from *S*. *officinarum*. In addition, six exclusive alleles (SCB381.0236, SCB436.0199, SCB312.0208, CV79.0228, CV106.0132, CV106.0078) derived from *S*. *spontaneum* were spread among twenty cultivars (CTC3, CTC10, CTC11, IAC87-3396, IAC91-1099, IACSP94-2101, IACSP95-3028, IACSP95-5000, RB835054, RB855536, RB925211, RB925345, RB928064, RB931530, SP832847, SP841201, SP8642, SP87344, SP891115, SP911049). None of the nineteen other species/exotic hybrids exclusive alleles were observed in cultivars.

Alleles CV38.0210, CV106.0135, SCB381.0244 and SCC423.0300 were absent in the basic germplasm (S3 Table) and hence, were exclusive among twelve cultivars (CTC13, CTC14, CTC17, IACSP94-2101, RB925268, SP775181, SP801836, SP803280, SP813250, SP842025, SP855077 and SP87396).

Regarding to the gene diversity values (S2 Table), *S*. *spontaneum* had the highest genetic diversity (D) estimate value (0.900) followed by other species/exotic hybrids (0.898), *S*. *officinarum* (0.883), and Brazilian commercial cultivars (0.873).

### Population differentiation and genetic structure

The degree of the genetic differentiation estimate (${\hat{\Phi }}_{ST}$) obtained between the Brazilian commercial cultivars and the basic germplasm was 0.179 (P<0.001), which means that 17.91% of the total variation found by the SSRs is distributed between these two groups, while 82.09% is within them. The genetic differentiation estimate between Brazilian commercial cultivars and *S*. *officinarum* accessions (${\hat{\Phi }}_{ST}$ = 0.211; P<0.001) was slightly lower than that observed between Brazilian commercial cultivars and *S*. *spontaneum* (${\hat{\Phi }}_{ST}$ = 0.216; P<0.001). The smallest ${\hat{\Phi }}_{ST}$ value was found when comparing *S*. *officinarum* and *S*. *spontaneum* accessions (${\hat{\Phi }}_{ST}$ = 0.127; P<0.001) (Table 3).

**Table 3:** Analysis of molecular variance (AMOVA) for predefined groups.

Groups	Source of Variation	d.f.	Sum of squares	Variance components	Percentage of variation
**Cultivars and Basic germplasm**	Among population	1	437.195	6.175	17.91
	Within populations	135	3821.972	28.311	82.09
	Total	136	4259.168	34.486	
	Fixation Index (${\hat{\Phi }}_{ST}$_1_): 0.179*				
**Cultivars and *S*. *officinarum***	Among population	1	278.349	7.259	21.09
	Within populations	101	2742.699	27.155	78.91
	Total	102	2021.049	34.415	
	Fixation Index (${\hat{\Phi }}_{ST}$_2_): 0.211*				
**Cultivars and *S*. *spontaneum***	Among population	1	412.637	8.515	21.64
	Within populations	110	3392.113	30.837	78.36
	Total	111	3804.750	39.352	
	Fixation Index (${\hat{\Phi }}_{ST}$_3_): 0.216*				
***S*. *officinarum* and *S*. *spontaneum***	Among population	1	187.674	5.755	12.70
	Within populations	51	2017.232	39.554	87.30
	Total	52	2204.906	45.309	
	Fixation Index (${\hat{\Phi }}_{ST}$_4_): 0.127*				

d.f.: degrees of freedom.

*P<0.001.

**Fig 2:**
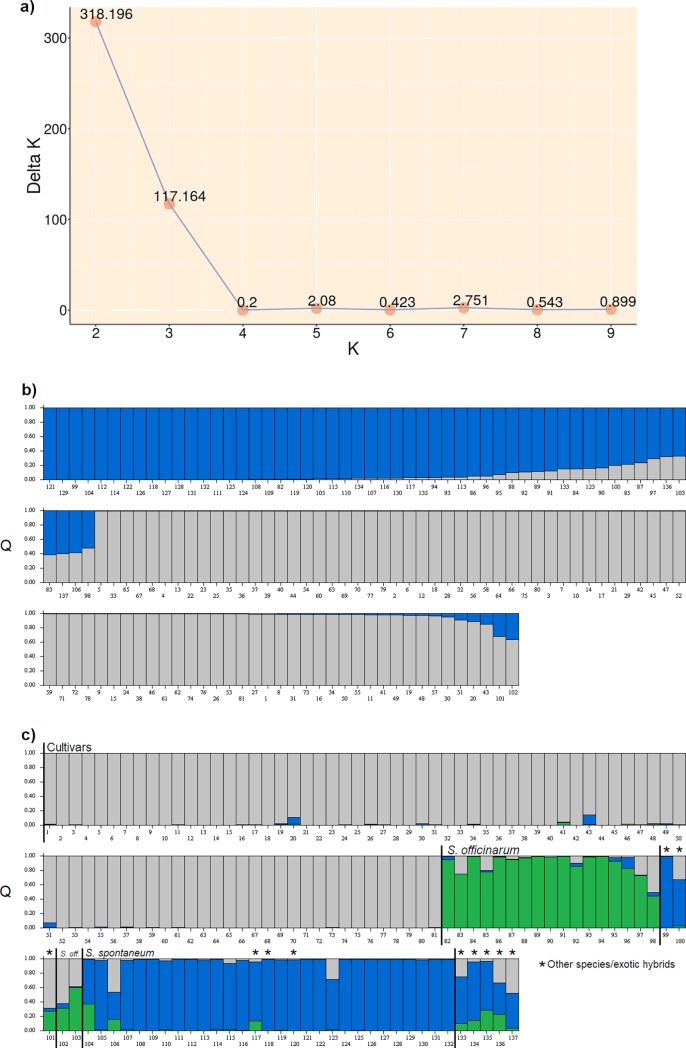
Clustering analysis. (a) Best k analysis showing k values from 2 to 9 (10 suppressed); (b) Membership proportion (Q) of each genotype: two distinct and almost homogeneous groups distinguishing individuals from 1 to 81 (cultivars, grey color) and 82 to 137 (basic germplasm, blue color) for k = 2; (c) three distinct groups, the first composed by 81 cultivars (grey color) and the remaining genotypes differing into two other groups comprised of the main species *S*. *officinarum* (green color) and *S*. *spontaneum* (blue color) for k = 3.

According to the structure analysis, 10 cluster subsets resulted for every input k value, which were submitted to Structure Harvester and CLUMPAK’s Best K pipeline. The best k value was 2 (ΔK = 318.196, Fig 2A), suggesting that the 137 genotypes can be divided into two groups (Fig 2B) corresponding to the Brazilian cultivars and basic germplasm. The second most relevant k value is 3 (ΔK = 117.164), suggesting that the group formed by the basic germplasm can be subdivided according to their main species (*S*. *officinarum* and *S*. *spontaneum*), capturing most of the data structure in three groups (Fig 2C). Bar colors denote the membership proportion for each genotype.

### Genetic dissimilarity and phylogenetic analysis

The number of markers used in this research was sufficient to estimate the pair-wise genetic dissimilarity with an acceptable level of accuracy. For all the 285 markers, the CV associated with the estimates of similarities was 6.86%, and thus were under the threshold previously established (10%). In fact, around 140 markers would be sufficient to obtain a CV average estimate around 10% (S2 Fig).

The average dissimilarity values within the cultivars, *S*. *officinarum*, *S*. *spontaneum*, and other species/exotic hybrids was 0.176, 0.206, 0.248, and 0.285, respectively. The average dissimilarity values between the cultivars and *S*. *officinarum* was 0.247, while for between the cultivars and *S*. *spontaneum* it was 0.277, and between the cultivars and accessions from other species/exotic hybrids it was 0.272. In relation to the two main sugarcane species, the average dissimilarity between *S*. *officinarum* and *S*. *spontaneum* accessions was 0.279. The average dissimilarity between the accessions from other species/exotic hybrids and *S*. *officinarum* was 0.282, and with *S*. *spontaneum* it was 0.278 (Table 4).

**Table 4:** Average dissimilarities between predefined groups according to DARwin extreme values function.

	Cultivars	*Saccharum officinarum*	*Saccharum spontaneum*	Other species/ exotic hybrids
**Cultivars**	0.176	-	-	-
***Saccharum officinarum***	0.247	0.206	-	-
***Saccharum spontaneum***	0.277	0.279	0.248	-
**Other species/exotic hybrids**	0.272	0.282	0.278	0.285

When comparing the average dissimilarity values between the commercial cultivars and each single basic germplasm accession, MIDAZ (*S*. *officinarum* x *S*. *barberi)* was the accession most genetically similar (0.203) to the commercial cultivars and CHUNNEE the most divergent (0.326) (S4 Table).

We observed a low dissimilarity value (0.077) between IAC87-3396 and SP87344, which are full sibs from the cross CO740 and SP701143, for example.

**Fig 3:**
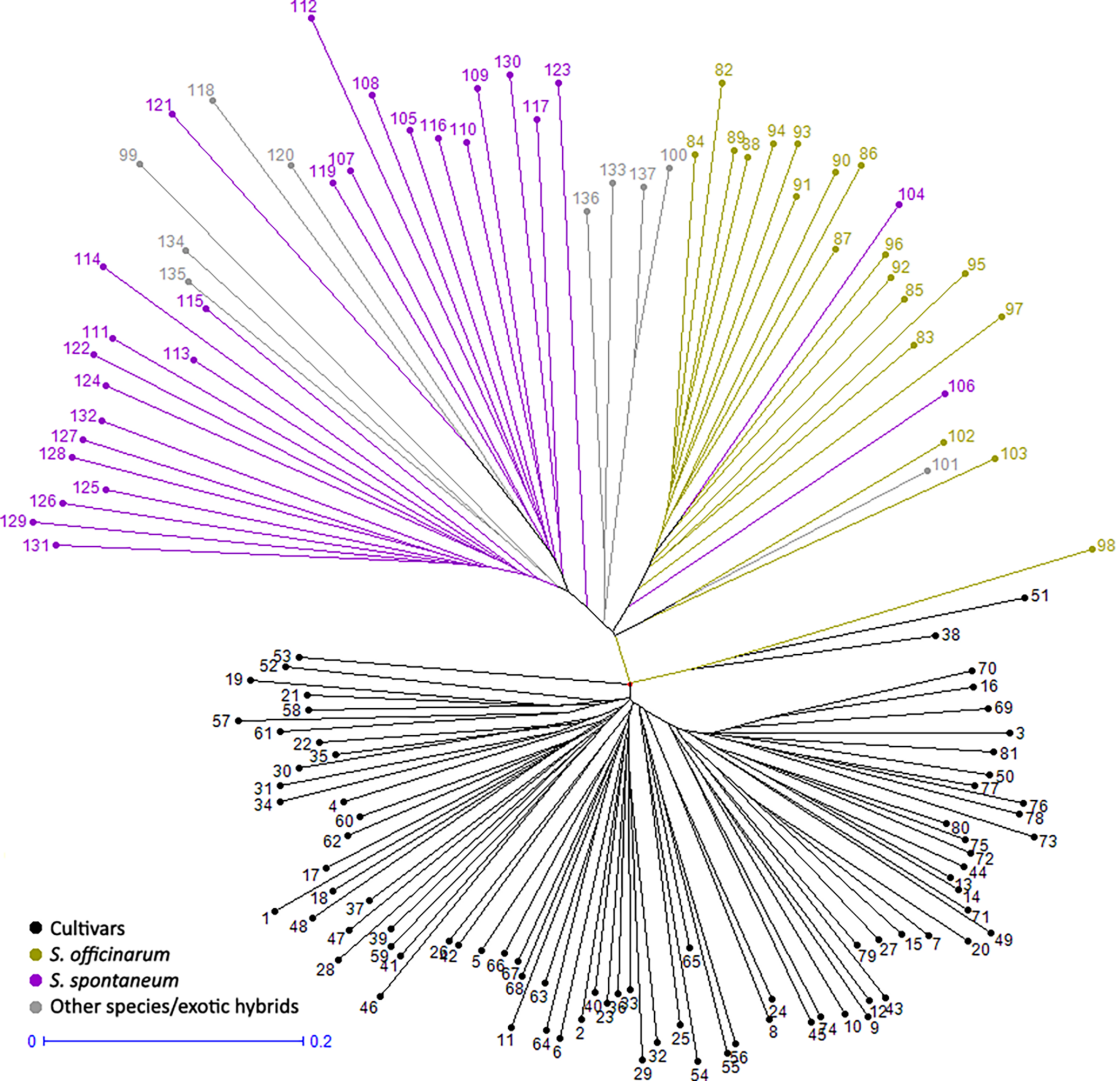
Phylogenetic tree analysis. Two distinct major clusters: on the bottom of the image the cultivars grouped in one well defined cluster; on the top of the image, accessions are grouped according to its main species. Other species/exotic hybrids are spread within this second group according to their parenthood (Method: Unweighted Neighbor-Joining).

The phylogenetic tree revealed the prevalence of two major groups (cultivars and basic germplasm) which agrees with the K = 2 value (two subpopulations) pointed to by the software Structure. In addition, the accessions were mainly clustered according to their species (*S*. *officinarum* and *S*. *spontaneum*), with the accessions from other species/exotic hybrids spread among these two groups suggesting the existence of three groups (Fig 3).

**Fig 4:**
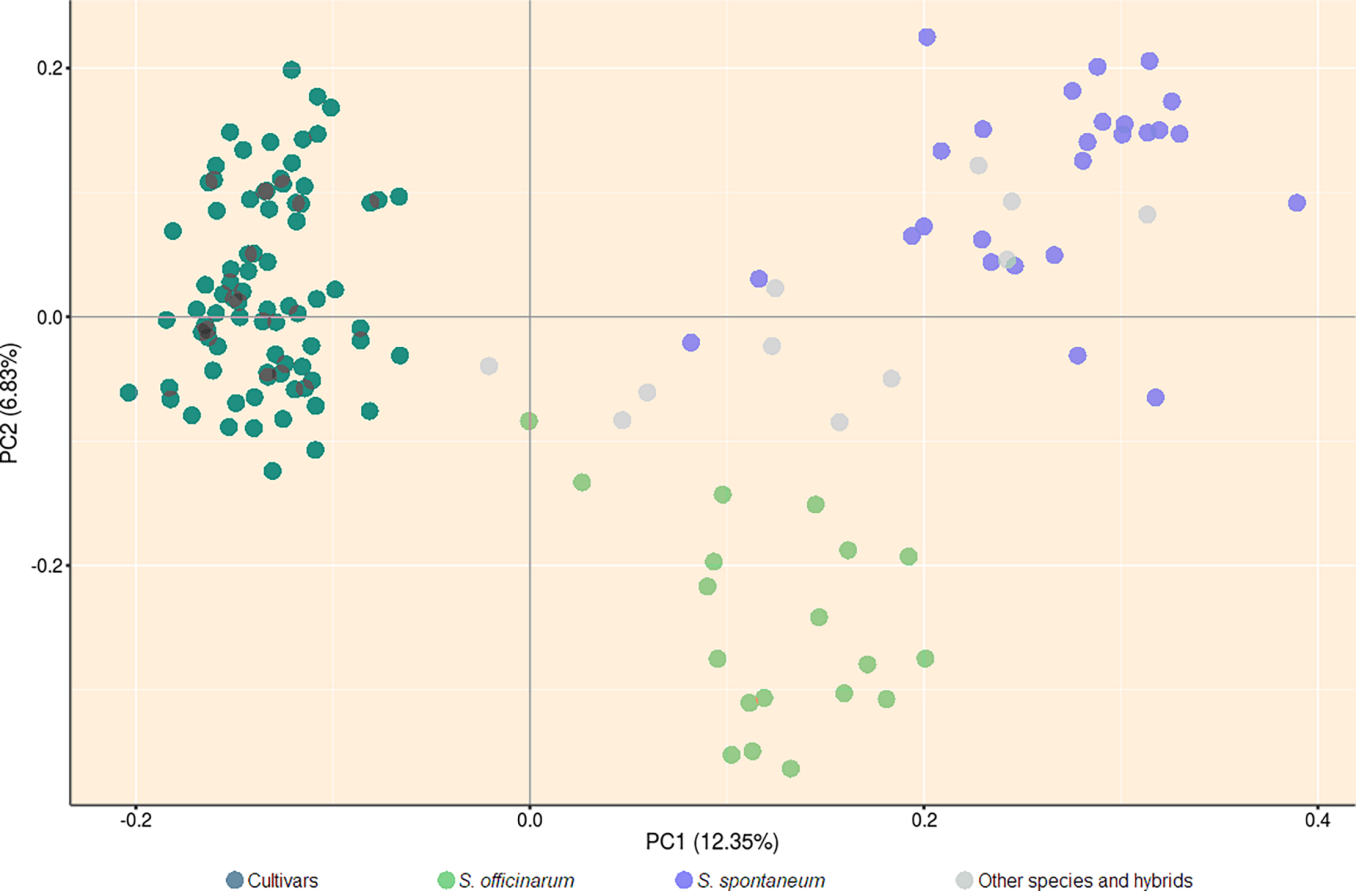
Principal Coordinate Analysis of sugarcane accessions based on dissimilarity matrix (1-Jaccard), between pairs of genotypes.

Together, the first two principal components (PCs) explained 19.18% of the total variability expressed among genotypes, based on dissimilarity measurement (Fig 4). According to the first PC, cultivars are grouped in a cluster isolated from the others. Looking at the second PC, *S*. *officinarum* accessions form a distinct group, while *S*. *spontaneum* and the other species/exotic hybrids tend to cluster together. On the other hand, it seems that there is a slight differentiation between these two groups, once *S*. *spontaneum* accessions are concentrated at the first quadrant of the graphic.

## Discussion

### Efficiency of the SSR primer set for sugarcane germplasm management and cultivar identification

Undoubtedly, the availability of an efficient and adequate size set of SSR primer pairs has a great impact for routine management of sugarcane germplasm and cultivar identification in a cost-effective way. Several indexes are available to evaluate primer pair efficiency for polymorphisms at the molecular level [21,22,28,47]. In general, the indexes used here to measure primer effectiveness showed high values for either all the genotypes or only the Brazilian cultivar panel. This indicates that a large amount of genetic information was captured by these 12 SSR primer pairs across this diverse sugarcane group.

Microsatellites allow the amplification of different allele types at a locus. Due to the sugarcane’s high ploidy level and consequent heterozygosity, more than two types of alleles per locus in a single individual can be observed and the “banding patterns” reveal the different allele types of a locus defined by one SSR primer pair, although the allele dosage or number of copies cannot be determined [20,41,44]. Therefore, a considerable number of alleles are observed on a gel line for a single individual with a single SSR primer pair. In fact, the average number of alleles obtained with the 12 SSR primer set was promising compared to those reported by Nayak et al. (2014) [10] in which 209 alleles were produced by 36 SSR primer pairs screened over 1,002 accessions from the World Collection of Sugarcane and Related Grasses (WCSRG) and by Pan (2010) [66] which obtained 144 alleles derived from 21 SSR primer pairs over a *Saccharum* spp. molecular database.

The high number of alleles obtained by the 12 SSR primer set also positively impacted PIC and Resolving Power (Rp). Most of the PIC values were above 0.7 for either the total accessions or the cultivars. The Resolving Power, which reveals the ability of a given primer pair to distinguish between genotypes [47], was high for half of the SSR primer pairs and most of them were able to distinguish more than 90% of the total evaluated accessions.

### Allelic richness and exclusive alleles

Due to its large ecogeographical distribution, *S*. *spontaneum* is considered the most diverse species in the *Saccharum* genus [67,68]. This species presented the highest number of alleles across the 12 microsatellite loci. In addition, of the total number of putative exclusive alleles detected in the basic germplasm, the greatest contribution was from *S*. *spontaneum* (63%), as compared to *S*. *officinarum* (6.5%) and other species/exotic hybrids (31%).

Cultivars encompassed more than half of the total alleles occurring in the basic germplasm showing a considerable representation of its allelic richness. However, a reasonable number of putative exclusive alleles derived from the basic germplasm (41.8%) was not inherited by the Brazilian commercial cultivars. In fact, a very limited number of accessions were used in the early interspecific crosses to develop the first commercial sugarcane cultivars [13,69]. Only 19 *S*. *officinarum*, two *S*. *spontaneum* and one *S*. *sinense* clones were extensively used to form the genetic base of modern sugarcane during the first interspecific hybridizations [12]. This genetic proportion appears to have been maintained with little additional genetic variation across generations of breeding programs, resulting in the narrow genetic base of current commercial cultivars and increasing the *S*. *officinarum* contribution [70].

Of the few putative-species-origin exclusive alleles within the Brazilian commercial cultivars, only one of four (25%) and six of thirty-nine (15.4%) were derived from *S*. *officinarum* and *S*. *spontaneum*, respectively. This could be a result of the nobilization process, as almost 80% of the commercial cultivar genome is from *S*. *officinarum*, indicating that most of the alleles present in the Brazilian commercial cultivars are shared with *S*. *officinarum*. On the other hand, the proportion of *S*. *spontaneum* exclusive alleles found in the Brazilian commercial cultivars seems to be close to the *S*. *spontaneum* genome contribution (around 10–15%) expected for commercial sugarcane cultivars [9].

This result also suggests that *S*. *spontaneum* had a significant contribution to the genetic variability of commercial cultivars used in Brazil. Aitken et al. (2006) [71], assessing the genetic diversity though AFLP markers in a collection of sugarcane cultivars and elite parents from the Australian breeding program, also reported that the variation observed for cultivars was attributed in large part to the *S*. *spontaneum* chromosomes.

We also reported the presence of putative exclusive alleles derived from commercial cultivars, suggesting that either the basic germplasm sampled in our work did not encompass the whole genetic pool used in the prior breeding programs, or that new alleles in commercial cultivars could have emerged over time as a result of DNA polymerase slippage or unbalanced crossing-over, which are mechanisms that lead to insertions or deletions of one or more repeats [72,73].

### Genetic structure of Brazilian commercial cultivars

The genetic differentiation index (${\hat{\Phi }}_{ST}$) proposed by Excoffier et al. (1992) [74] is considered analogous to Wright’s fixation index, F_ST_. According to Wright (1978) [75], F_ST_ values between 0 to 0.05 signals little population differentiation while values in the 0.05–0.15 and 0.15–0.25 ranges, respectively, indicate moderate and strong genetic differentiation. The genetic differentiation value between the Brazilian commercial cultivars and *S*. *officinarum* was slightly lower (21.1%) than that observed between commercial cultivars and *S*. *spontaneum* (21.6%), and in turn, these values reflect a strong genetic differentiation between the commercial cultivars and the two main *Saccharum* species. This strong genetic differentiation between Brazilian commercial cultivars and these main species reflects the previously mentioned founder effect, combined with the genetic drift effect, as a small number of these two progenitor species’ clones contributed to the current genetic base of commercial sugarcane cultivars. Moreover, for the Brazilian cultivars, there is a high number of shared common parents among them, which also contributes to an increase in the genetic differentiation between commercial cultivars and their ancestor species.

Despite the limited genotypes employed in the first breeding programs, the sugarcane genetic base has not narrowed as much as had been suspected [54], especially given the high ploidy and genetic diversity of the major ancestral species. On the other hand, the high genetic differentiation between the Brazilian cultivars and *S*. *officinarum* and *S*. *spontaneum* signals that considerable genetic variability can be incorporated in the current genetic breeding pool by exploiting both species, especially *S*. *spontaneum* for energy cane development.

Moderate genetic differentiation was obtained between *S*. *officinarum* and *S*. *spontaneum* exposing a genetic relationship between these two species. As pointed out by Aitken and McNeil (2010) [35], based on Stevenson (1965) [76], it was hypothesized that sugarcane evolved from a primitive form of *S*. *spontaneum* in the Himalaya foothills of northern India, from which, as a consequence of evolutionary forces such as selection, migration, and introgression, two centers of diversity, respectively for *S*. *spontaneum* in India and *S*. *robustum* in New Guinea had resulted. However, it is well accepted that *S*. *officinarum* has emerged from *S*. *robustum* in New Guinea by domestication of this later species [77].

### Genetic dissimilarity and cluster analysis

The four groups previously established (Cultivars, *S*. *officinarum*, *S*. *spontaneum*, and other species/exotic hybrids) was confirmed by the PCoA results. The commercial cultivars were assigned to a separate cluster, as expected, since most of them share the same parents and were bred to meet the high demand for sugar and ethanol production. The *S*. *officinarum* accessions have outstanding features such as high sugar production, low fiber content, and thick stalks, among others, which differs from related species. In accordance with the phenotypic differentiation, divergence at the molecular level was observed between *S*. *officinarum* accessions and the other species/exotic hybrids, justifying the formation of an isolated cluster composed by genotypes of this species. *S*. *spontaneum*, and the other species/exotic hybrids are more similar, and some overlap was shown between these groups. However, there is a slight trend toward separation among them (Fig 4). All these results agree with those obtained by the other methods applied in this study.

As expected, the highest average dissimilarity (0.285) was observed within the accessions of the other species/exotic hybrids group while the lowest (0.176) within the cultivars group. The average dissimilarity obtained within cultivars was 1.2 and 1.4 times lower than that within *S*. *officinarum* and *S*. *spontaneum*, respectively. This result can have practical applications for sugarcane breeding programs focusing on energy cane, once the choice of more divergent parents (S4 Table) amplifies the genetic variability in the progeny and also the possibility to maximize the range of sugar-to-fiber ratios and hence the selection for Type I (higher fiber content and lower sucrose content) and Type II (fiber content higher than Type I and marginal sugar content) of energy canes [78,79].

The average dissimilarity within cultivars implies that on average the commercial Brazilian cultivars shared 82% of the genomic regions assessed with SSRs. This is the first report in which a very large number of currently planted Brazilian cultivars was investigated at the molecular level, giving a more complete picture of their genetic relationship.

The pairwise dissimilarities of Brazilian cultivars ranged from 0.077 (IAC87-3396 x SP87344) to 0.258 (CTC2 x SP801836), and was lower than that observed in SSR assays by Pocovi and Mariotti (2015) [80] with 66 cultivars from the INTA (National Institute of Agricultural Technology) Sugarcane Germplasm Bank, in Argentina, ranging from 0.079 to 0.651. Hemaprabha et al. (2005) [81] assessed the genetic diversity of 54 commercial cultivars developed in India by Sequence Tagged Microsatellite Sites (STMS), a SSR-based technique, reporting 238 possible combinations between the fifty-four materials with lower similarity (<0.650) out of 1,431 possible crossings, which points to a very low-diversity genetic pool. You et al. (2013) [82] used SSR markers to evaluate the genetic structure and diversity of 115 sugarcane clones bred in China. Genetic similarity ranged from 0.725 to 1.000, revealing a very high level of uniformity among those cultivars, strongly enforcing the usage of new parents to broaden the Chinese sugarcane genetic base. A subsequent study [83] encompassing some of these 115 cultivars from the earlier study, within a group of 181 Chinese cultivars, reported that the usage of these novel parents in crossings may have an important role in broadening the genetic base of sugarcane bred in China. These results highlight the need to broaden the Brazilian sugarcane genetic pool, perhaps in the direction of available unexploited basic germplasm.

Clustering and phylogenetic analyses revealed the parenthood among the total genotypes evaluated. They tend to cluster according to their species and genealogy, forming consistent groups in agreement with the pedigree data available. However, some discrepancies were observed. Two *S*. *spontaneum* individuals, GLAGAH and IN8488, were grouped within *S*. *officinarum*. In fact, despite its classification as *S*. *spontaneum*, GLAGAH is a possible natural hybrid between *Saccharum* species. Although IN8488 is considered a *S*. *spontaneum* clone, the membership proportion showed high identity correlation with *S*. *officinarum*, suggesting that it probably is also a hybrid. POJ2878 and KASSOER are tightly clustered in the phylogenetic analysis. In addition, the structure analysis revealed almost the same membership proportion of the three groups in both individuals, revealing an already expected low genetic dissimilarity (0.0947) because POJ2878 is a direct F2 descendant of KASSOER [11].

## Supporting information

S1 Fig Gel images of three SSR regions. Polyacrylamide electrophoresis assays generated high quality images allowing accurate genotyping.
(TIF)

S2 Fig Curve of the coefficient of variation (CV%) according to the different numbers of markers considered. (TIF)

S1 Table Genotypes used in this study. Names with respective species, female and male genitors, and origin.
(DOCX)

S2 Table Allele survey. Number of alleles, exclusive alleles, species exclusive alleles found in commercial cultivars, absent alleles in commercial cultivars and gene diversity.
(DOCX)

S3 Table Exclusive putative alleles within groups plus allele occurrence and origin. (DOCX)

S4 Table Basic germplasm mean dissimilarities between each accession and the group of cultivars. (DOCX)
