# Supplementary figures and images for: Unraveling the genetic structure of Brazilian commercial sugarcane cultivars through microsatellite markers
Source: PLoS One. 2018 Apr 23;13(4):e0195623. doi: 10.1371/journal.pone.0195623 (PMC5912765; doi:10.1371/journal.pone.0195623)

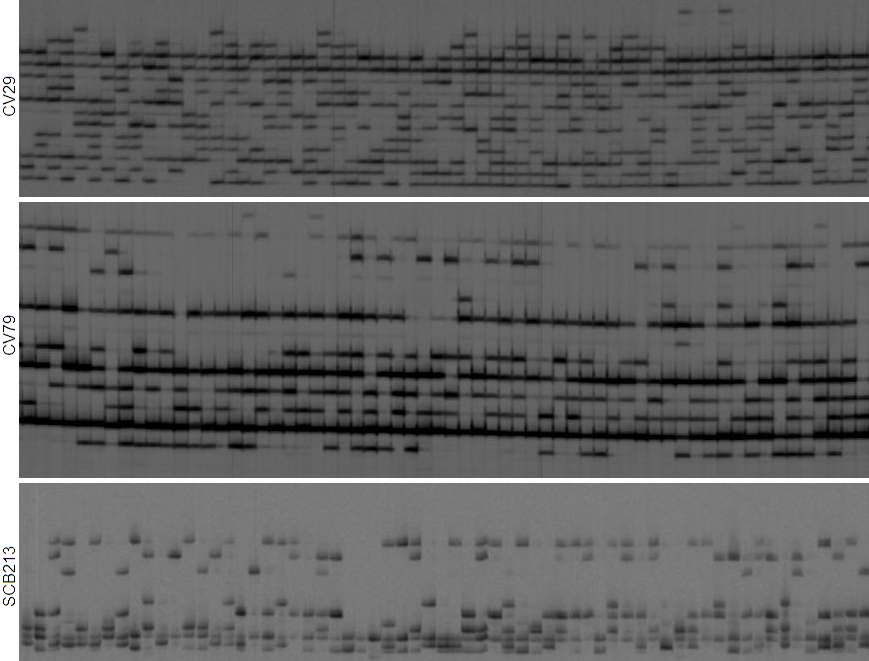

Supplement: S1 Fig — Polyacrylamide electrophoresis assays generated high quality images allowing accurate genotyping. (TIF) [file pone.0195623.s001.tif]

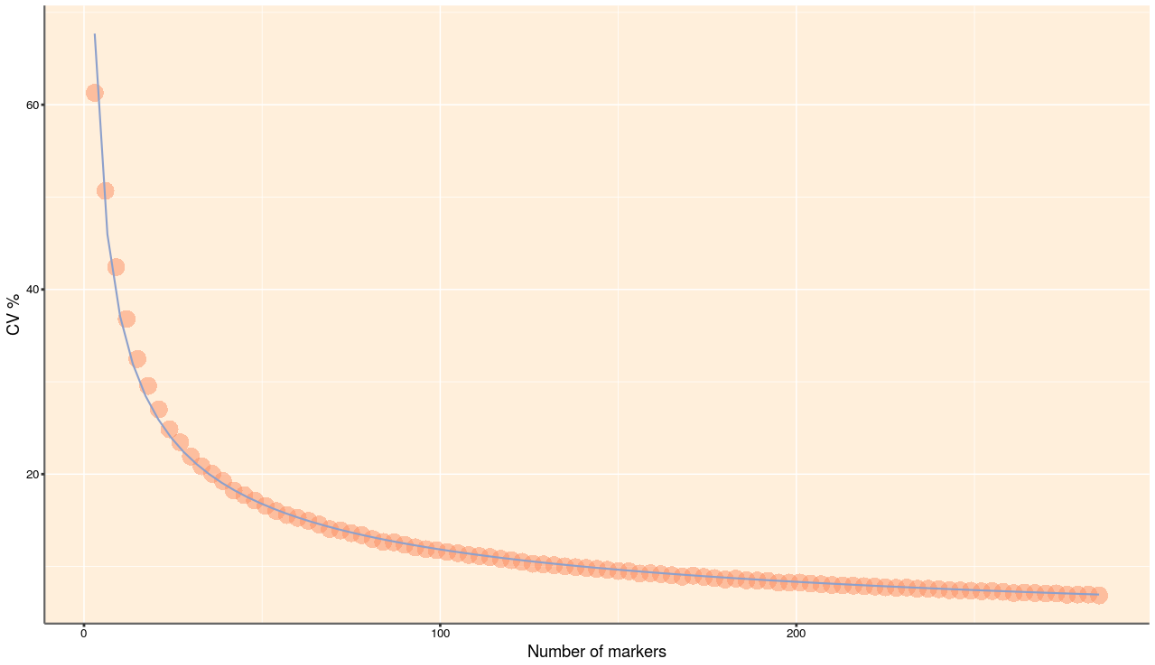

Supplement: S2 Fig — (TIF) [file pone.0195623.s002.tif]
