# Supplementary material for: Unraveling the genetic structure of Brazilian commercial sugarcane cultivars through microsatellite markers
Source: PLoS One. 2018 Apr 23;13(4):e0195623. doi: 10.1371/journal.pone.0195623 (PMC5912765; doi:10.1371/journal.pone.0195623)
Supplement: S1 Table — Names with respective species, female and male genitors, and origin. (DOCX) [file pone.0195623.s003.docx]

| **S1 Table. Genotypes used in this study.** Names with respective species, female and male genitors, and origin. | | | | | |
| --- | --- | --- | --- | --- | --- |
| **#** | **Genotype** | **Species** | **Female Genitor** | **Male Genitor** | **Origin** |
| **1** | SP87396 | *Saccharum* spp. hybrid | Q76 | ? | Brazil - CTC |
| **2** | SP842025 | *Saccharum* spp. hybrid | CO1007 | SP716180 | Brazil - CTC |
| **3** | SP903414 | *Saccharum* spp. hybrid | SP801079 | SP823544 | Brazil - CTC |
| **4** | SP801816 | *Saccharum* spp. hybrid | SP711088 | H575028 | Brazil - CTC |
| **5** | SP901638 | *Saccharum* spp. hybrid | SP784601 | ? | Brazil - CTC |
| **6** | SP841201 | *Saccharum* spp. hybrid | CO62175 | ? | Brazil - CTC |
| **7** | SP801842 | *Saccharum* spp. hybrid | SP711088 | H575028 | Brazil - CTC |
| **8** | SP813250 | *Saccharum* spp. hybrid | CP701547 | SP711279 | Brazil - CTC |
| **9** | SP87365 | *Saccharum* spp. hybrid | SP773067 | ? | Brazil - CTC |
| **10** | SP855077 | *Saccharum* spp. hybrid | H393633 | SP721861 | Brazil - CTC |
| **11** | SP8642 | *Saccharum* spp. hybrid | SP70-1143 | ? | Brazil - CTC |
| **12** | SP775181 | *Saccharum* spp. hybrid | HJ5741 | ? | Brazil - CTC |
| **13** | SP87344 | *Saccharum* spp. hybrid | CO740 | SP701143 | Brazil - CTC |
| **14** | SP845560 | *Saccharum* spp. hybrid | IAC52-326 | ? | Brazil - CTC |
| **15** | SP803280 | *Saccharum* spp. hybrid | SP711088 | H575028 | Brazil - CTC |
| **16** | SP811763 | *Saccharum* spp. hybrid | CO775 | NA5679 | Brazil - CTC |
| **17** | SP841431 | *Saccharum* spp. hybrid | H518029 | ? | Brazil - CTC |
| **18** | SP803480 | *Saccharum* spp. hybrid | Q73 | H611820 | Brazil - CTC |
| **19** | SP832847 | *Saccharum* spp. hybrid | HJ5741 | SP701143 | Brazil - CTC |
| **20** | SP801836 | *Saccharum* spp. hybrid | SP711088 | H575028 | Brazil - CTC |
| **21** | SP891115 | *Saccharum* spp. hybrid | CP731547 | ? | Brazil - CTC |
| **22** | SP86155 | *Saccharum* spp. hybrid | SP783081 | ? | Brazil - CTC |
| **23** | SP853877 | *Saccharum* spp. hybrid | H65606 | ? | Brazil - CTC |
| **24** | SP911049 | *Saccharum* spp. hybrid | SP803328 | SP813250 | Brazil - CTC |
| **25** | SP835073 | *Saccharum* spp. hybrid | SP711406 | SP711088 | Brazil - CTC |
| **26** | SP80185 | *Saccharum* spp. hybrid | BO17 | ? | Brazil - CTC |
| **27** | SP801520 | *Saccharum* spp. hybrid | H483166 | SP711088 | Brazil - CTC |
| **28** | CTC1 | *Saccharum* spp. hybrid | LCP8110 | ? | Brazil - CTC |
| **29** | CTC2 | *Saccharum* spp. hybrid | SP821176 | ? | Brazil - CTC |
| **30** | CTC3 | *Saccharum* spp. hybrid | SP7652 | ? | Brazil - CTC |
| **31** | CTC4 | *Saccharum* spp. hybrid | SP835073 | ? | Brazil - CTC |
| **32** | CTC5 | *Saccharum* spp. hybrid | SP821176 | ? | Brazil - CTC |
| **33** | CTC6 | *Saccharum* spp. hybrid | SP811763 | SP791416 | Brazil - CTC |
| **34** | CTC7 | *Saccharum* spp. hybrid | RTT8618 | ? | Brazil - CTC |
| **35** | CTC8 | *Saccharum* spp. hybrid | SP813493 | ? | Brazil - CTC |
| **36** | CTC9 | *Saccharum* spp. hybrid | SP831483 | ? | Brazil - CTC |
| **37** | CTC10 | *Saccharum* spp. hybrid | SP785495 | ? | Brazil - CTC |
| **38** | CTC11 | *Saccharum* spp. hybrid | SP843478 | ? | Brazil - CTC |
| **39** | CTC12 | *Saccharum* spp. hybrid | SP801842 | SP811747 | Brazil - CTC |
| **40** | CTC13 | *Saccharum* spp. hybrid | SP842029 | ? | Brazil - CTC |
| **41** | CTC14 | *Saccharum* spp. hybrid | SP801842 | SP775181 | Brazil - CTC |
| **42** | CTC15 | *Saccharum* spp. hybrid | SP842025 | ? | Brazil - CTC |
| **43** | CTC17 | *Saccharum* spp. hybrid | ? | ? | Brazil - CTC |
| **44** | IAC87-3396 | *Saccharum* spp. hybrid | CO740 | SP701143 | Brazil - IAC |
| **45** | IAC91-1099 | *Saccharum* spp. hybrid | RB785148 | ? | Brazil - IAC |
| **46** | IACSP94-4004 | *Saccharum* spp. hybrid | SP826108 | SP775181 | Brazil - IAC |
| **47** | IACSP94-2101 | *Saccharum* spp. hybrid | SP775181 | RB785148 | Brazil - IAC |
| **48** | IACSP94-2094 | *Saccharum* spp. hybrid | SP847017 | ? | Brazil - IAC |
| **49** | IAC86-2480 | *Saccharum* spp. hybrid | US71399 | ? | Brazil - IAC |
| **50** | IACSP95-3028 | *Saccharum* spp. hybrid | SP842189 | RB855156 | Brazil - IAC |
| **51** | IACSP95-5000 | *Saccharum* spp. hybrid | SP842066 | SP80185 | Brazil - IAC |
| **52** | IACSP93-3046 | *Saccharum* spp. hybrid | SP791011 | ? | Brazil - IAC |
| **53** | RB935744 | *Saccharum* spp. hybrid | RB835089 | RB765418 | Brazil - RIDESA |
| **54** | RB825336 | *Saccharum* spp. hybrid | H533989 | ? | Brazil - RIDESA |
| **55** | RB925211 | *Saccharum* spp. hybrid | RB855206 | ? | Brazil - RIDESA |
| **56** | RB925345 | *Saccharum* spp. hybrid | H591966 | ? | Brazil - RIDESA |
| **57** | RB925268 | *Saccharum* spp. hybrid | RB855511 | ? | Brazil - RIDESA |
| **58** | RB931530 | *Saccharum* spp. hybrid | ? | ? | Brazil - RIDESA |
| **59** | RB928064 | *Saccharum* spp. hybrid | SP701143 | ? | Brazil - RIDESA |
| **60** | RB93509 | *Saccharum* spp. hybrid | ? | ? | Brazil - RIDESA |
| **61** | RB835054 | *Saccharum* spp. hybrid | RB72454 | NA5679 | Brazil - RIDESA |
| **62** | RB855035 | *Saccharum* spp. hybrid | L6014 | SP701284 | Brazil - RIDESA |
| **63** | RB855536 | *Saccharum* spp. hybrid | SP701143 | RB72454 | Brazil - RIDESA |
| **64** | RB845197 | *Saccharum* spp. hybrid | RB72454 | SP701143 | Brazil - RIDESA |
| **65** | RB842021 | *Saccharum* spp. hybrid | ? | ? | Brazil - RIDESA |
| **66** | RB92579 | *Saccharum* spp. hybrid | RB755126 | RB72199 | Brazil - RIDESA |
| **67** | RB867515 | *Saccharum* spp. hybrid | RB72454 | ? | Brazil - RIDESA |
| **68** | RB865230 | *Saccharum* spp. hybrid | RB72454 | SP701143 | Brazil - RIDESA |
| **69** | RB855453 | *Saccharum* spp. hybrid | TUC717 | ? | Brazil - RIDESA |
| **70** | RB845257 | *Saccharum* spp. hybrid | RB72454 | SP701143 | Brazil - RIDESA |
| **71** | RB855563 | *Saccharum* spp. hybrid | TUC717 | SP701143 | Brazil - RIDESA |
| **72** | RB855113 | *Saccharum* spp. hybrid | SP701143 | RB72454 | Brazil - RIDESA |
| **73** | RB855463 | *Saccharum* spp. hybrid | ? | ? | Brazil - RIDESA |
| **74** | RB813804 | *Saccharum* spp. hybrid | ? | ? | Brazil - RIDESA |
| **75** | RB855036 | *Saccharum* spp. hybrid | RB72454 | SP701143 | Brazil - RIDESA |
| **76** | RB855511 | *Saccharum* spp. hybrid | ? | ? | Brazil - RIDESA |
| **77** | RB855546 | *Saccharum* spp. hybrid | SP701143 | RB72454 | Brazil - RIDESA |
| **78** | RB845210 | *Saccharum* spp. hybrid | RB72454 | SP701143 | Brazil - RIDESA |
| **79** | RB835486 | *Saccharum* spp. hybrid | L6014 | ? | Brazil - RIDESA |
| **80** | RB966928 | *Saccharum* spp. hybrid | RB855156 | RB815690 | Brazil - RIDESA |
| **81** | RB855156 | *Saccharum* spp. hybrid | RB72454 | TUC717 | Brazil - RIDESA |
| **82** | AJAX | *S. officinarum* | - | - | Fiji |
| **83** | BADILA | *S. officinarum* | - | - | Australia |
| **84** | BLACK BORNEO | *S. officinarum* | - | - | Indonesia |
| **85** | BOURBON SUR | *S. officinarum* | - | - | - |
| **86** | BRAVA DE PERICO | *S. officinarum* | - | - | - |
| **87** | CRIOULA | *S. officinarum* | - | - | - |
| **88** | CAIANA LISTRADA | *S. officinarum* | - | - | - |
| **89** | CAIANA ROXA | *S. officinarum* | - | - | - |
| **90** | CAIANA VERDADEIRA | *S. officinarum* | - | - | - |
| **91** | CANA ALHO | *S. officinarum* | - | - | - |
| **92** | CERAM RED | *S. officinarum* | - | - | India |
| **93** | GREEM GERMAN | *S. officinarum* | - | - | India |
| **94** | IJ76418RED | *S. officinarum* | - | - | Indonesia |
| **95** | KHAJURIA | *S. officinarum* | - | - | India |
| **96** | MUNTOKJAVA | *S. officinarum* | - | - | Indonesia |
| **97** | RP8 | *S. officinarum* | - | - | - |
| **98** | ENDOR | *S. officinarum* | - | - | Australia |
| **99** | CHUNNEE ² | *S. barberi* | - | - | India |
| **100** | KASSOER ¹ | *S. off.* x *S. spo.* | BLACKCHERI | *S. spontaneum* | Indonesia |
| **101** | MIDAZ ¹ | *S. off.* x *S. barberi* | TROJAN | VESTA | - |
| **102** | NG2121 | *S. officinarum* | - | - | Papua New Guinea |
| **103** | PITU | *S. officinarum* | - | - | - |
| **104** | GLAGAH | *S. spontaneum* | - | - | Indonesia |
| **105** | HOLES1 | *S. spontaneum* | - | - | - |
| **106** | IN8488 | *S. spontaneum* | - | - | Indonesia |
| **107** | IN81101 | *S. spontaneum* | - | - | Indonesia |
| **108** | IS76196 | *S. spontaneum* | - | - | USA - Hawaii |
| **109** | M.MOENTAI | *S. spontaneum* | - | - | Indonesia |
| **110** | NG26011 | *S. spontaneum* | - | - | Papua New Guinea |
| **111** | PCAV8413 | *S. spontaneum* | - | - | Philippines |
| **112** | SES260 | *S. spontaneum* | - | - | India |
| **113** | SES365 | *S. spontaneum* | - | - | India |
| **114** | SES196 | *S. spontaneum* | - | - | India |
| **115** | SH301 | *S. spontaneum* | - | - | India |
| **116** | KRAKATAU | *S. spontaneum* | - | - | Indonesia |
| **117** | US851008 ¹ | *S. spontaneum x ?* | - | - | Taiwan |
| **118** | NG5712 ² | *S. robustum* | - | - | Papua New Guinea |
| **119** | IND81170 | *S. spontaneum* | - | - | India |
| **120** | UK3739 ² | *Saccharum spp.* | - | - | - |
| **121** | IK76006 | *S. spontaneum* | - | - | USA - Hawaii |
| **122** | IN8109M2 | *S. spontaneum* | - | - | Indonesia |
| **123** | IN81014 | *S. spontaneum* | - | - | Indonesia |
| **124** | PCAV84-6-9 | *S. spontaneum* | - | - | Philippines |
| **125** | SES35379 | *S. spontaneum* | - | - | India |
| **126** | SES073-38 | *S. spontaneum* | - | - | India |
| **127** | SES234-2 | *S. spontaneum* | - | - | India |
| **128** | SES208-1 | *S. spontaneum* | - | - | India |
| **129** | PURPLE-32 | *S. spontaneum* | - | - | - |
| **130** | COIMBATORE | *S. spontaneum* | - | - | India |
| **131** | GLAGAH KLOET | *S. spontaneum* | - | - | Indonesia |
| **132** | 51NG26 | *S. spontaneum* | - | - | Papua New Guinea |
| **133** | GANDACHENI ¹ | *S. barberi X ?* | - | - | India |
| **134** | 57NG2 ² | *S. robustum* | - | - | Papua New Guinea |
| **135** | FIJI10 ² | *Miscanthus* | - | - | Fiji |
| **136** | FIJI15 ² | *Miscanthus* | - | - | Fiji |
| **137** | POJ2878 ¹ | *Saccharum* spp. hybrid | EK28 | POJ2364 | Indonesia |
| CTC: Sugarcane Technology Center (*Centro de Tecnologia Canavieira*); IAC: Agronomic Institute of Campinas (*Instituto Agronômico de Campinas*); RIDESA: Interuniversity Network for the Development of the Sugarcane Sector (*Rede Interuniversitária para o Desenvolvimento do Setor Sucroenergético*); ¹ Exotic hybrids; ² Accessions representing other species. | | | | | |
