# Supplementary material for: Unraveling the genetic structure of Brazilian commercial sugarcane cultivars through microsatellite markers
Source: PLoS One. 2018 Apr 23;13(4):e0195623. doi: 10.1371/journal.pone.0195623 (PMC5912765; doi:10.1371/journal.pone.0195623)
Supplement: S2 Table — Number of alleles, exclusive alleles, species exclusive alleles found in commercial cultivars, absent alleles in commercial cultivars and gene diversity. (DOCX) [file pone.0195623.s004.docx]

| **Number of alleles** | **Total** | **%**  **(of 285)** | **Gene diversity** |
| --- | --- | --- | --- |
| Total population | 285 | - | - |
| Commercial cultivars | 166 | 58.25% | 0.873 |
| *S. officinarum* | 186 | 65.26% | 0.883 |
| *S. spontaneum* | 246 | 86.32% | 0.900 |
| Other species/exotic hybrids | 215 | 75.44% | 0.898 |
| **Exclusive Alleles** | **Total** | **%**  **(of 285)** | **%**  **(of 62)** |
| Commercial cultivars exclusive alleles | 4 | 1.40% | - |
| Exclusive alleles from basic germplasm: | 62 | 21.7% | - |
| *S. officinarum* exclusive alleles | 4 | 1.40% | 6.5% |
| *S. spontaneum* exclusive alleles | 39 | 13.68% | 63% |
| Other species/exotic hybrids exclusive alleles | 19 | 6.67% | 31% |
| **Species** **exclusive alleles found in commercial cultivars** | **Total** | **%**  **(of 285)** | **%**  ***(exclusive**  **alleles)** |
| *S. officinarum* exclusive alleles found in commercial cultivars | 1 | 0.35% | 25% (1/4) |
| *S. spontaneum* exclusive alleles found in commercial cultivars | 6 | 2.11% | 15.4%.(6/39) |
| Other species/exotic hybrids exclusive alleles found in commercial cultivars | 0 | 0.00% | - |
| **Absent alleles in commercial cultivars** | **Total** | **%**  **(of 285)** |  |
| Absent in commercial cultivars | 119 | 41.8% | - |
|  | **Total** | **%**  **(of 55)** | **%**  ***(exclusive**  **alleles)** |
| **Putative exclusive alleles absent in comercial cultivars:** | 55 |  |  |
| Absent in commercial cultivars, exclusive of *S. officinarum* | 3 | 5.45% | 75% (3/4) |
| Absent in commercial cultivars, exclusive of *S. spontaneum* | 33 | 60% | 84.6% (33/39) |
| Absent in commercial cultivars, exclusive of other species/exotic hybrids | 19 | 34.5% | 100% (19/19) |

Table S2: Allele survey: number of alleles, exclusive alleles, species exclusive alleles found in commercial cultivars, absent alleles in commercial cultivars and gene diversity.

* % obtained in relation to the respective species total exclusive alleles.
