# Supplementary material for: Unraveling the genetic structure of Brazilian commercial sugarcane cultivars through microsatellite markers
Source: PLoS One. 2018 Apr 23;13(4):e0195623. doi: 10.1371/journal.pone.0195623 (PMC5912765; doi:10.1371/journal.pone.0195623)
Supplement: S3 Table — (DOCX) [file pone.0195623.s005.docx]

| Table S3: Exclusive putative alleles within groups plus allele occurence and origin. | | | |
| --- | --- | --- | --- |
| **Origin** | **Putative Exclusive Alleles** | **Cultivars carrying the allele** | **Putative species origin (accessions)** |
| **Cultivars** | SCB381.0244 | SP775181; SP803280; SP801836 |  |
|  | SCC423.0300 | RB925268 |  |
|  | CV38.0210 | SP87396; SP842025; SP855077; SP775181; CTC13; CTC14; CTC17;  IACSP94-2101 |  |
|  | CV106.0135 | SP813250 |  |
| ***S. officinarum*** | SCB436.0186 | SP841431; CTC10; RB865230 | CREOULA; CAIANALISTRADA |
| ***S. spontaneum*** | SCB381.0236 | SP841201; SP891115; SP911049; CTC11; RB931530; RB835054; RB855536 | HOLES1; IN8109M2; SES07338 |
|  | SCB436.0199 | SP87344; SP832847; CTC10; IAC87-3396; IAC91-1099; RB928064 | SH301 |
|  | SCB312.0208 | CTC11; IACSP94-2101; IACSP95-3028; IACSP95-5000; RB925211; RB925345 | NG26011; IN8109M2; SES2081; GLAGAHKLOET; 51NG26 |
|  | CV79.0228 | SP8642 | PURPLE32 |
|  | CV106.0132 | CTC03 | SES260 |
|  | CV106.0078 | CTC03 | SH301; KRAKATAU; SES2342 |
