# Supplementary material for: Unraveling the genetic structure of Brazilian commercial sugarcane cultivars through microsatellite markers
Source: PLoS One. 2018 Apr 23;13(4):e0195623. doi: 10.1371/journal.pone.0195623 (PMC5912765; doi:10.1371/journal.pone.0195623)
Supplement: S4 Table — (DOCX) [file pone.0195623.s006.docx]

Table S4: Mean dissimilarities between each basic germplasm accession and the group of cultivars.

| **Accession** | **Mean Dissimilarity** | **Accession** | **Mean Dissimilarity** |
| --- | --- | --- | --- |
| MIDAZ | 0.203 | GANDACHENI | 0.264 |
| NG2121 | 0.211 | IND81170 | 0.266 |
| ENDOR | 0.222 | AJAX | 0.270 |
| BOURBONSUR | 0.224 | NG26011 | 0.272 |
| BADILA | 0.226 | IJ76418RED | 0.273 |
| IN8488 | 0.238 | MUNTOKJAVA | 0.274 |
| CANAALHO | 0.242 | NG5712 | 0.276 |
| IN81101 | 0.242 | SES260 | 0.278 |
| BLACKBORNEO | 0.243 | UK3739 | 0.278 |
| GREENGERMAN | 0.243 | 57NG2 | 0.281 |
| KRAKATAU | 0.245 | KASSOER | 0.281 |
| IN81014 | 0.246 | PCAV8413 | 0.284 |
| CAIANALISTRADA | 0.247 | IK76006 | 0.286 |
| CERAMRED | 0.247 | GLAGAH | 0.286 |
| PITU | 0.249 | FIJI15 | 0.287 |
| CREOULA | 0.250 | SES196 | 0.288 |
| SES365 | 0.251 | 51NG26 | 0.290 |
| CAIANAROXA | 0.251 | SES2081 | 0.290 |
| KHAJURIA | 0.251 | PCAV8469 | 0.291 |
| US85108 | 0.252 | FIJI10 | 0.291 |
| BRAVADEPERICO | 0.252 | SES35379 | 0.293 |
| CAIANAVERDADEIRA | 0.257 | M.MOENTAI | 0.294 |
| POJ2878 | 0.259 | SES2342 | 0.297 |
| HOLES1 | 0.259 | GLAGAHKLOET | 0.298 |
| COIMBATORE | 0.260 | IN8109M2 | 0.302 |
| IS76196 | 0.260 | PURPLE32 | 0.302 |
| RP8 | 0.260 | SES07338 | 0.313 |
| SH301 | 0.263 | CHUNNEE | 0.326 |
